# Serum amyloid A (SAA) concentration after training sessions in Arabian race and endurance horses

**DOI:** 10.1186/1746-6148-9-91

**Published:** 2013-05-01

**Authors:** Anna Cywinska, Lucjan Witkowski, Ewa Szarska, Antoni Schollenberger, Anna Winnicka

**Affiliations:** 1 Department of Pathology and Veterinary Diagnostics, Faculty of Veterinary Medicine, Warsaw University of Life Sciences - SGGW, Warsaw, Poland; 2 Laboratory of Veterinary Epidemiology and Economic Faculty of Veterinary Medicine, Warsaw University of Life Sciences – SGGW, Warsaw, Poland; 3 Military Institute of Hygiene and Epidemiology, Warsaw, Poland

**Keywords:** Serum amyloid A, Race horses, Endurance horses, Training

## Abstract

**Background:**

Serum amyloid A (SAA) is the major acute phase protein in horses. Its concentration increases in various pathologies but also in response to prolonged, strenuous effort. The purpose of this study was to establish whether routine race and endurance training produces changes in the SAA level in Arabian horses. Additionally, the differences between SAA response in experienced endurance horses and endurance horses that were beginning their career were investigated.

**Results:**

There were no changes in SAA concentrations after race training and endurance training in experienced horses. In horses that were beginning their endurance training, exercise produced an increase in SAA level as compared with rest level.

**Conclusion:**

In Arabians, the SAA concentration seems to be a good indicator of endurance training but is useless in race training. The routine training of experienced horses, which were prepared for long distance rides, did not promote any changes in the SAA level. In contrast, a significant increase in the SAA concentration was observed in horses that were beginning their endurance training and were only prepared for moderate distance rides and underwent the same effort. Further research is needed to elucidate whether this difference reflects too heavy training or adaptation to an increasing workload. Additionally, the adaptation to long distance rides in Arabians may include a reduced acute phase response.

## Background

Serum amyloid A (SAA) is the major acute phase protein in horses [1-7]. An increase in SAA concentration in the blood reflects the presence of an acute phase reaction (APR), which poses the first non-specific systemic response to any type of disturbances in homeostasis caused by infectious and non-infectious factors. In horses, of all the acute phase proteins, SAA is the most sensitive indicator of pathology and increases in clinical and experimental inflammatory conditions, including aseptic arthritis and laminitis [4,8,9], surgical trauma [4,8,10-13], septicaemia and focal inflammations [2,3], grass sickness [14], pneumonia, fever [15], colic [14,16], bacterial infections caused by *Rhodococcus* spp. and *Streptococcus equi*[9,10,17,18], viral infections caused by equine influenza virus and equine herpes virus [8,10,19], enteritis and diarrhoea of various origin [4], and also in response to physical stress caused by transportation [20]. During disease, the serum concentration of SAA increases quickly and with large amplitude, 100-fold to 1000-fold, with the level of change reflecting the amount of tissue damage [2-8,13,17,21,22]; this increase indicates the presence of pathology but not the exact diagnosis. The serum SAA concentration may also reflect subclinical states and the severity of infections [17,19]. The role of SAA is still not completely understood because it has numerous effects on leukocyte function, synthesis of inflammatory mediators, lipid transportation to inflamed tissue and the induction of enzymes that are responsible for degradation of extracellular matrix. Thus, SAA may have roles during many phases of the inflammatory response [5-7]. Measuring the SAA concentration is simple and, although non-specific, is a useful tool in preventive medicine programs that can help determine whether further tests are needed [3,7,21]. It is also useful for monitoring the health status of race and performance horses, which helps avoid the stress of transportation or competition in sick animals [7,20] and may help estimate whether the horse is fit for training [3]. Similarly, the SAA level together with the white blood cell count may be considered as useful parameters to improve equine transporting conditions [20].

Despite the typical acute phase reaction that occurs during inflammatory conditions, the increase in acute phase proteins concentrations may also be produced by exertion, most likely due to glycogen depletion in working muscles and skeletal muscle damage [23]. This exercise-induce acute phase reaction - APR has been described after long lasting strenuous exercise in human, dogs and horses [23-26]. The reaction pattern differs from APR in inflammation and varies among species. In the horses, this reaction is observed after long, but not moderate, distance endurance rides and is characterised by a marked increase in SAA but not other acute phase proteins levels [26]. We have also reported that horses with pre-competition SAA levels higher than 1 mg/L failed to complete their long distance rides [25]. Thus, measuring the serum SAA concentration before entering a competition may indicate whether the horse is in poor condition, including subclinical disorders, overtraining or slight injuries that may worsen with exertion and lead to elimination from the ride [25]. Other authors have reported an increase in SAA concentration after moderate treadmill exercise during an experimental study on the effect of an antioxidative compound on exercise-induced muscle damage [27]. This observation is in agreement with Liburt’s et al. findings that the level of proinflammatory and acute phase-related cytokines, including IL-1, TNF-α and IL-6 (in the muscles), increased after an incremental exercise test in unfit Standardbreds [28]. Horohov et al. confirmed that proinflammatory cytokines are elevated 2 hours after routine race training [29]. Sporting events induce various types of reaction due to different environmental factors and stress levels, different type of exercise and horse-dependent factors [30], which may also affect the exercise-induced acute phase response.

The aim of this study was to compare the changes in SAA concentrations in horses during anaerobic (race) and aerobic (endurance) training and, in the latter, the variations in horses that were undergoing regular training or were beginning their endurance career. To avoid the impact of stress resulting from competing and to maintain constant environmental conditions in the groups, the horses were tested during training and not during competitions.

## Methods

### Horses and training

Arabian horses were chosen because they are used in both disciplines. Twenty-two privately owned Arabians, divided into 3 groups (A – horses in race training; B – horses in endurance training, with little training experience but similarly aged as the race horses; and C – horses experienced in endurance training), were included in the study. All procedures were approved by the Third Warsaw Local Ethics Committee for Animal Experimentation at SGGW and the owners of the horses. The horses were dewormed and vaccinated at the proper time and housed in two training centres, Sluzewiec Race Track in Warsaw and Champion Stable Training Centre in Ciosny, based on whether they were undergoing race or endurance training, respectively; thus, the environment was the same for the race and endurance horses. They were fed the diet recommended for each discipline and consumed daily oats (5.5 kg for race horses, 2 kg for endurance horses in groups B and C) and a complete high quality commercial feed (0.5-1 kg for race horses, 1 kg for endurance horses in group B, 2–2.5 kg for endurance horses in group C), distributed over three feedings. Additionally, 6 – 7 kg of standard meadow hay was administered *ad libitum.* The animals were confirmed to be healthy based on a clinical examination before and after exertion (performed by a qualified veterinarian according to the rules accepted for equine disciplines) and haematological analysis (to exclude pathological conditions), and they were selected by their training status, as evaluated by the trainer.

Group A consisted of 10 horses (6 stallions and 4 mares), 3 to 5 years of age. The horses underwent regular race training, with daily training sessions, when the speed of the fast gallop gradually increased before race season and then was held constant (during training sessions performed from May to October). They exercised together, participated in races and were classified in general handicap. They were examined before and after training session performed in August, under clear weather with temperatures of 18-20°C, on a sand track (Sluzewiec Race Track in Warsaw). Training sessions included warm-up walking and trotting with the rider, followed by cantering and fast gallop (45.44 ± 4.16 km/h) for 800 m, and 30 min of exercise with a horse walker.

Group B included 5 horses (3 stallions and 2 mares), 5 to 7 years of age, that began their endurance training, had not competed yet and were being prepared for moderate distances (up to 80 km) rides.

Group C consisted of 7 horses (3 stallions, 2 mares and 2 geldings), 7 to 15 years of age, that engaged in regular endurance training. The horses participated in long distance (120 and 160 km) endurance rides in previous seasons and were being prepared for such competitions.

Both groups of endurance horses were examined before and after training session similar to competition, while they exercised together, performed once a month. Examination was performed in May, under clear weather with temperatures of 20-22°C, at the routine 50 km loop. Training included walking, trotting and galloping; time and speed were individually predicted for each horse by heart rate, which was monitored with a heart rate monitor (RS800 on Polar Equine Wearlink W.I.N.D – Polar Electro Oy, Finland), and the average speed was 12.94 ± 2.17 km/h.

### Blood samples

Blood samples were obtained by jugular venipuncture before and after training sessions, when horses were in stalls. The first (rest) samples were collected around 6 a.m., and the second (post-exercise) samples were collected 8.30 a.m. from race horses and 5 p.m. from endurance horses. Samples were aspirated into 20 ml syringe and immediately transferred into EDTA-3K tubes for haematological tests and plain tubes for serum analyses. In race horses, lactate (LA) concentrations were determined immediately by ejecting a drop of whole blood onto a single-use lactate strip (Accusport, Roche, Germany). The tubes were kept in the refrigerator (+4°C) and analysed within 6 hours of collection. Routine haematological parameters, including haematocrit (HCT), haemoglobin (HGB), the red blood cell (RBC) count and the white blood cell (WBC) count, were counted with an automated haematology analyser (Abacus, France). Differential counts were determined manually from smears by counting 200 cells, and the neutrophil to lymphocyte ratio (N:L) was calculated. The tubes without anticoagulant were centrifuged at 4380 *g* for 5 minutes, and the serum was aspirated, immediately frozen, and stored at −20°C until analysis. Serum samples were used for measurement of SAA, total protein (TP) concentrations and creatine phosphokinase (CPK) activity in endurance horses. Creatine phosphokinase activity was assayed by the kinetic method (Pointe Scientific, USA). Total protein levels were determined using Biuret Reagent (Pointe Scientific, USA). SAA levels were measured using a double sandwich ELISA (Phase Serum Amyloid A Assay, Tridelta Ltd., Ireland).

### Statistical analysis

Statistical procedures, means and standard errors of mean were computed using STATISTICA 6.0 for Windows. The results are expressed as the mean ± standard error of the mean (SEM). The Kolmogorov-Smirnov test indicated that the data were not normally distributed*.* The differences in SAA concentrations, leukograms (WBC, neutrophil and lymphocyte numbers and N:L ratio) and erythrograms (RBC, HGB and HCT) were evaluated between groups and in response to exercise in each group.

The results before and after the training sessions (response to exercise) in each group were compared with Wilcoxon matched pairs test with Bonferroni correction in case of leukogram and erythrogram changes. Statistical comparisons using the Kruskal-Wallis test were performed between the results obtained before and after exercise in groups. A p ≤ 0.05 (after Bonferroni correction p ≤ 0.017 for erythrograms and p ≤ 0.013 for leukograms) was considered significant.

## Results

**Figure 1:**
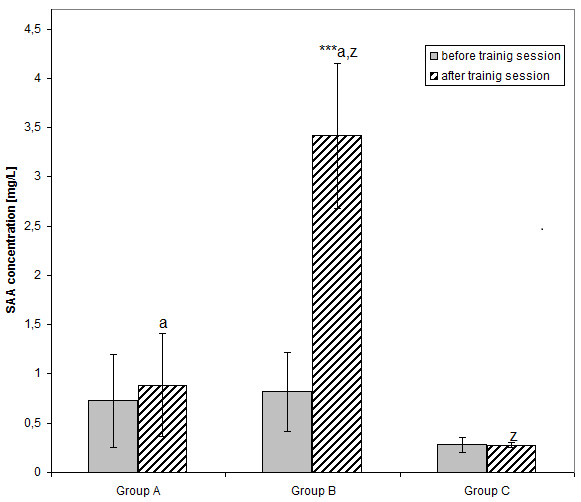
**Serum amyloid A (SAA) concentration in the horses before and after training sessions.** Group A – race horses, Group B – inexperienced endurance horses, Group C – experienced endurance horses. Significant differences were observed between the following groups: before and after training sessions in each group: *p ≤ 0.05, **p ≤ 0.01, ***p ≤ 0.001. groups A and B: ^a^p ≤ 0.05, ^b^p ≤ 0.01, ^c^p ≤ 0.001. groups A and C: ^d^p ≤ 0.05, ^e^p ≤ 0.01, ^f^p ≤ 0.001. groups B and C: ^x^p ≤ 0.05, ^y^p ≤ 0.01, ^z^p ≤ 0.001.

Both race and endurance efforts were intense enough to produce biochemical changes that were typical for each discipline: the LA concentration increased in race horses(from 1.78 ± 0.02 to 4.9 ± 0.61 mmol/L), and the CPK activity increased in endurance horses (from 235.12 ± 13.43 to 640.47 ± 214.15 U/L in inexperienced endurance horses and from 280.1 ± 68.87 to 390.28 ± 133.46 U/L in experienced endurance horses). All haematological parameters (Table 1) and SAA concentrations (Figure 1) determined before and after training sessions in all horses varied within the normal ranges for equine species [31].

**Table 1:** Haematological parameters in the horses before and after training sessions

	**Group A (race horses)**		**Group B (inexperienced endurance horses)**		**Group C (experienced endurance horses)**	
	**Before**	**After**	**Before**	**After**	**Before**	**After**
WBC [×10^9^/L]	8.4 ± 0.24	9.61 ± 0.64^a, d^	8.86 ± 0.44	12.88 ± 1.4*^, a^	7.55 ± 1.58	11.53 ± 1.44*^, d^
RBC [×10^12^/L]	9.23 ± 0.18^b, f^	11.45 ± 0.76***^, e^	8.08 ± 0.16^b^	8.7 ± 0.17*^b^	7.15 ± 0.79^f^	8.06 ± 0.56^e^
HGB [g/dL]	14.74 ± 0.35^a^	18.11 ± 0.9***^, b, d^	13.66 ± 0.27^a^	14.56 ± 0.28^b^	12.52 ± 0.84	14.44 ± 0.81^d^
HCT [%]	36.45 ± 0.72^a^	45.12 ± 2.52***^b^	33.76 ± 0.6^a^	36.74 ± 0.75**^b^	34.24 ± 2.34	38.16 ± 2.66
NEU [×10^9^/L]	4.93 ± 0.3	5.66 ± 0.44^b^	4.8 ± 0.22	10.76 ± 1.35**^b^	4.19 ± 0.35	7.8 ± 1.22
LYM [×10^9^/L]	3.3 ± 0.18	3.74 ± 0.3^a^	3.64 ± 0.38	1.99 ± 0.29*^a^	3.1 ± 0.67	2.94 ± 0.45
N:L	1.56 ± 0.15	1.59 ± 0.18^c, d^	1.43 ± 0.15	6.09 ± 1.2***^c^	1.5 ± 0.17	2.89 ± 0.5^d^

WBC – white blood cell count, RBC – total red blood cell count, HGB – haemoglobin concentration, HCT – packed cell volume, NEU – total number of neutrophils, LYM – total number of lymphocytes, N:L – neutrophil to lymphocyte ratio.

Significant differences were observed between the following groups:

Before and after training sessions in each group:*p ≤ 0.05, **p ≤ 0.01, ***p ≤ 0.001.

Groups A and B: ^a^p ≤ 0.05, ^b^p ≤ 0.01, ^c^p ≤ 0.001.

Groups A and C: ^d^p ≤ 0.05, ^e^p ≤ 0.01, ^f^p ≤ 0.001.

Groups B and C: ^x^p ≤ 0.05, ^y^p ≤ 0.01, ^z^p ≤ 0.001.

### Changes after training in the groups

In race horses (group A), the training effort resulted in significant (p ≤ 0.001) increases in all erythrogram parameters. In inexperienced endurance horses (group B), the training session produced significant increases in the SAA concentration (p ≤ 0.001), RBC and HCT values (less so than in race horses, p ≤ 0.05 and p ≤ 0.01, respectively, when compared to rest values) and changes in leukogram parameters (increases in WBC, p ≤ 0.05; neutrophils, p ≤ 0.01; and the N:L ratio, p ≤ 0.001 and a decrease in lymphocytes, p ≤ 0.05). In group C, only the WBC increase in response to the training session was significant (p ≤ 0.05), and the other parameters exhibited great variation among individual horses.

### Comparison of pre- and post-training values between groups

Before the training sessions, no significant differences were noted between the groups of endurance horses (B and C). The pattern of changes in groups B and C was similar, and no significant differences in erythrogram and leukogram parameters were noted between groups B and C after the training. Only the post-exercise SAA concentration was significantly (p ≤ 0.001) higher in group B compared with group C.

Differences before exercise were observed between groups A and B; race horses displayed a higher RBC count (p ≤ 0.01) and HCT and HGB values (p ≤ 0.05 in both cases). Before training, groups A and C differed only in RBC, which was higher (p ≤ 0.001) in the race horses. After the training session, groups A and B differed in all tested parameters. SAA concentration, WBC, neutrophils and the N:L ratio were significantly (p ≤ 0.05, p ≤ 0.05, p ≤ 0.01 and p ≤ 0.001, respectively) higher in endurance horses (group B). In race horses (group A), the erythrogram parameters RBC, HGB and HCT were significantly (p ≤ 0.01 in all cases) higher and the lymphocyte numbers were significantly (p ≤ 0.05) lower compared with the endurance horses (group B). After exercise, groups A and C differed in RBC and HGB values, which were significantly higher in race horses (p ≤ 0.01 and p ≤ 0.05, respectively), and the WBC count and N:L ratio, which were significantly higher (p ≤ 0.05 in both cases) in endurance horses (group C).

## Discussion

Proper training depends on the discipline and should lead to metabolic changes and physiological adaptations that improve performance during competitions. Some authors [32] have shown that there is a relationship between haematological changes and performance that can help modify the training schedule to achieve the desired performance. Different adaptations are required across disciplines, and various haematological changes develop in trained horses, which were observed in our study as the differences between the pre-exercise haematological parameters in race and endurance horses. General improvement occurs with time, but certain parameters (e.g., heart rate, HCT, and LA concentrations) change during and after each training session and help estimate whether the workload is proper for the horse. In this study, we analysed basic haematological parameters, which changed depending on the type of training and the training experience of the endurance horses. The additional parameter SAA was chosen due to our previous finding that horses with an SAA level higher that 1 mg/L did not complete their long distance rides [25]; however, when the SAA level should be determined before competitions is unknown. Thus, it seemed helpful to characterise the changes in SAA concentrations after routine training.

The patterns of haematological changes in response to exercise were typical and reflected the type of exertion. According to previous reports, the increase in erythrogram parameters in race horses may reflect the requirement for higher oxygen uptake per time unit at faster speeds. These changes result from the release of splenic erythrocytes due to spleen contraction under the influence of catecholamines [33]. During endurance training, haematological changes reflect exercise-induced stress, which depends on increased catecholamine and cortisol levels; these hormones promote the mobilisation of splenic erythrocytes and the release of the neutrophil marginal pool and lymphocyte to tissues and organs, resulting in neutrophilic leukocytosis and an increased N:L ratio [34-38]. Exercise-induced haematological changes have been widely described, and some of them are used to monitor training progress [32].

In this study, changes in SAA concentration were investigated for the first time after training. Long distance competitions were previously shown to produce exercise-induced acute phase reaction, which manifested as a 10-fold or greater increase in SAA concentration. After moderate distance rides, the SAA level increases approximately 2-fold, which was not sufficient to be interpreted as exercise-induced APR [26]. Generally, the effort during training sessions was not as great as during competitions, so we did not expect a high increase; however, it was not clear if any reaction occurred.

In race horses, the SAA concentration did not change after training, but large variations among individual horses were noted. The lack of changes could be expected because post-exercise samples were collected approximately 2.5 hours after the rest samples, which was most likely too early to detect an increase in the SAA concentration. Inflammatory stimuli produce an increase in SAA concentration within 12–16 hours [9,13], and this increase persists for at least 48 hours [5,9,11,13,19]. Non-inflammatory stimulation, e.g., transportation stress, also induced a reaction that includeds an increase in SAA level between 4 and 12 hours; however, a significant rise was detected at 48 hours [20]. Even within 4–5 hours after a single inflammatory stimulus, SAA concentration may become measurable [4]. The kinetics of reaction likely depends on the type of stimulation and may vary among individuals [8], but the SAA concentration generally increases rapidly within a few hours [3,10]. The increased SAA level is induced by IL-6, which increases in blood 30 min – 1.5 hours after stimulation. Thus, 2.5 hours appears to be too soon to detect significant changes. However, the horses underwent the same training the previous day, so if any exercise-induced increase occurred, it would have persisted until the following morning, and the level of SAA at rest would have been higher. Thus, considering the characteristics of race training, which include daily training sessions that pose similar potential to stimulate SAA production every 24 hours, using the SAA concentration to monitor race training is useless. However, it is still valuable for detecting pathology.

Although not significant, there were some differences between the values measured before and after exercise. These differences may result from intra-assay imprecision, which was reported previously [39] for SAA values lower than 50 mg/L, particularly lower than 1 mg/L. In our study, SAA concentrations in race horses were slightly higher than 1 mg/L, but both, variations among individuals and analytical imprecision may cause the differences between mean values observed before and after the training.

In endurance horses, the time of sampling was sufficient to detect changes in the SAA concentration after exertion (as observed in group B). In experienced horses, the SAA concentration was very low, and there was no evidence of an exercise-induced acute phase reaction or any change in the SAA level after 50 km training session. The workload, however, was sufficient to produce increases in CPK activity and WBC count. Thus, routine training promotes changes that lead to adaptation to exercise; however, this training is not harmful and does not stimulate an acute phase reaction. Therefore, any increases in SAA concentrations in experienced endurance horses should be considered only as a result of overtraining or pathology (including subclinical states). SAA may be a useful indicator of the endurance horse’s condition when preparing to start if it is measured several days before a competition, when there is time for rest or additional care for horses with increased SAA levels.

In inexperienced endurance horses, the SAA concentration increased significantly by approximately 4-fold, indicating a systemic reaction. Although this increase was not high enough to indicate disease, it might suggest that this training session posed a strong and even potentially harmful effort. On the other hand, it may represent the normal onset of adaptation to the training routine. At the beginning of their endurance careers, the horses do not compete in long distance competitions, and their training is generally less intense than the training of experienced endurance horses, so training of the same intensity poses different effort for experienced and inexperienced horses. The increase in post-exercise CPK activity in the inexperienced group was not as high as it was after heavy endurance effort. Thus, the SAA concentration may be more sensitive than CPK activity as an indicator of the horse’s status after heavy training. We cannot exclude the hypothesis that stimulation of a systemic reaction is part of the adaptation to training. In humans who are actively involved in various types of sports, repeated training sessions result in an increased concentration of C-reactive protein, which is the main acute phase protein in humans [40]. However, the detailed kinetics of adaptation to training have not been investigated in humans or horses. In addition, the systemic reaction in endurance horses that are beginning their careers may normally be stimulated by heavier training, but it depends on the horse’s status and predisposition whether it reflects adaptation or a potentially harmful effect with additional required care. In the current study, the increase in SAA concentration after exercise was evident in all inexperienced endurance horses. While this observation seems important, further research that includes an analysis of SAA profiles in individual horses is needed to evaluate the use of SAA measurements for monitoring endurance training.

Considering the characteristics of endurance training, including sessions of various intensity during general monthly programs, the SAA concentration can be measured without limitations mentioned in race horses. In endurance, heavier training is not preceded by similar effort the day or two days before, so an increase in SAA level would directly reflect the changes produced by the actual training session.

In general, the SAA concentrations determined in our study were low in all groups. The reference range is very wide (0–20 mg/L [7,33,41]), but Beaufort Cottage Laboratories [41] recommends 1.3 mg/L for healthy adult horses, which is in agreement with the values reported by other authors and our previous studies [13,25,26]. Thus, the horses examined in the current study matched the criteria for healthy horses. Only the increased SAA level in inexperienced endurance horses after exertion may suggest an unfavourable reaction; however, no clinical abnormalities were noted. The values at rest did not differ significantly among groups but were higher in inexperienced endurance (group B) and race (group A) horses, and large variations among individuals occurred in these groups, in contrast with the experienced endurance horses (group C). This fact may result from long lasting regular training. A similar phenomenon has been reported in human athletes who trained regularly [42]. The levels of C-reactive protein (the main acute phase protein in humans) were lower in athletes who trained regularly than in untrained controls and depended on the discipline. In horses, regular race training affects the haptoglobin and fibrinogen concentrations after 60–80 days [43]. Generally, in the case of training in human athletes, adaptation to exercise leads to a reduced inflammatory response [44], and a similar tendency has also been observed in thoroughbred race horses that undergo regular training [29]. The long-term effect of endurance training on inflammatory capacity has not been tested in horses; however, a similar effect cannot be excluded. This hypothesis is in agreement with the low and similar SAA concentration observed among individual endurance horses in our study.

## Conclusion

In conclusion, our study has shown that measuring SAA concentration may be helpful for monitoring endurance training but is useless in race horses. In endurance horses that are beginning their career, the SAA level measured after heavier training increased, which may be interpreted as either an adaptive response or an indication of too heavy, potentially harmful training. Further research, including the analyses of SAA profiles in individual horses, is needed to understand this phenomenon. In experienced endurance horses, the levels of SAA did not change after routine training and were generally lower than reported by other authors in different breeds. This fact suggests that the adaptation to long-lasting endurance training may be associated with a reduced acute phase response in Arabian horses; however, the mechanism of this phenomenon remains to be elucidated.

## Competing interests

The authors declare that they have no competing interests.

## Authors’ contributions

AC: designed the study, performed analysis, analyzed data and wrote the paper. LW: contributed in laboratory analysis. ES: contributed in samples collection and data analysis. AS: supervised and edited the manuscript. AW: supervised the project. All authors read and approved the final manuscript.
